# Tyrosine Kinase Signaling in Cancer Metabolism: PKM2 Paradox in the Warburg Effect

**DOI:** 10.3389/fcell.2018.00079

**Published:** 2018-07-24

**Authors:** Elizabeth K. Wiese, Taro Hitosugi

**Affiliations:** ^1^Department of Molecular Pharmacology and Experimental Therapeutics, Mayo Clinic, Rochester, MN, United States; ^2^Molecular Pharmacology and Experimental Therapeutics Graduate Program, Mayo Clinic Graduate School of Biomedical Sciences, Mayo Clinic, Rochester, MN, United States; ^3^Division of Oncology Research, Mayo Clinic, Rochester, MN, United States

**Keywords:** the Warburg Effect, tyrosine kinases, PKM2, lactate, pyruvate kinase

## Abstract

The Warburg Effect, or aerobic glycolysis, is one of the major metabolic alterations observed in cancer. Hypothesized to increase a cell's proliferative capacity via regenerating NAD^+^, increasing the pool of glycolytic biosynthetic intermediates, and increasing lactate production that affects the tumor microenvironment, the Warburg Effect is important for the growth and proliferation of tumor cells. The mechanisms by which a cell acquires the Warburg Effect phenotype are regulated by the expression of numerous oncogenes, including oncogenic tyrosine kinases. Oncogenic tyrosine kinases play a significant role in phosphorylating and regulating the activity of numerous metabolic enzymes. Tyrosine phosphorylation of glycolytic enzymes increases the activities of a majority of glycolytic enzymes, thus promoting increased glycolytic rate and tumor cell proliferation. Paradoxically however, tyrosine phosphorylation of pyruvate kinase M2 isoform (PKM2) results in decreased PKM2 activity, and this decrease in PKM2 activity promotes the Warburg Effect. Furthermore, recent studies have shown that PKM2 is also able to act as a protein kinase using phosphoenolpyruvate (PEP) as a substrate to promote tumorigenesis. Therefore, numerous recent studies have investigated both the role of the classical and non-canonical activity of PKM2 in promoting the Warburg Effect and tumor growth, which raise further interesting questions. In this review, we will summarize these recent advances revealing the importance of tyrosine kinases in the regulation of the Warburg Effect as well as the role of PKM2 in the promotion of tumor growth.

## Introduction

Approximately 90 years ago, Otto Warburg described the phenotype he observed in cancer cells where he noted that cancer cells display increased glucose consumption and increased lactate production regardless of oxygen availability (Warburg, 1956). This upregulation of glycolysis, coined the Warburg Effect or aerobic glycolysis, is a common phenotype in cancer; approximately 70% of 2,000,000 cancer tissues examined display high expression of the genes related to the Warburg Effect as compared to the more than 2,000,000 examined normal tissues (Altenberg and Greulich, 2004). Cancer cells possess the ability to proliferate rapidly, survive under hypoxic conditions, avoid immune surveillance, and metastasize; alterations in cellular metabolism are necessary to promote each of these characteristics (Hanahan and Weinberg, 2011). While it may seem counterintuitive for cancer to upregulate a less ATP and energy producing pathway as compared to the mitochondrial oxidative phosphorylation pathway, there are numerous advantages to the Warburg Effect. The Warburg Effect allows for rapid regeneration of NAD^+^ from NADH by lactate dehydrogenase A (LDHA), the ability to survive in hypoxic environments due to decreased dependence on oxidative phosphorylation, and increased glycolytic biosynthetic intermediates to support macromolecule biosynthesis (Gatenby and Gillies, 2004; Lunt and Vander Heiden, 2011). The increase in lactate production has also been proposed to aid in avoiding immune surveillance as well as acidifying the tumor microenvironment to aid in metastasis (Gillies et al., 2002; Gottfried et al., 2006; Fischer et al., 2007). Thus, there is a growth and proliferative advantage for cancer cells that display the Warburg Effect, and additional mechanisms by which a cell acquires this metabolic phenotype continue to be the focus of numerous studies.

Aberrant oncogene expression that drives oncogenesis also alters cellular metabolism and can promote the Warburg Effect. One mechanism by which oncogenes promote the Warburg Effect is via transcriptional regulation of glycolytic enzymes. Numerous genes coding for glycolytic enzymes contain consensus motifs for the binding of HIF-1 or c-myc (Kim et al., 2004). Therefore, overexpression of c-myc or HIF-1 results in increased transcription and increased gene expression of multiple glycolytic enzymes and, therefore, the subsequent increase in glycolytic activity observed with the Warburg Effect.

In addition to transcriptional regulation, post-translational modifications also alter the protein localization, enzymatic activity, or stability of glycolytic enzymes to promote the Warburg Effect. Aberrant kinase activity is one of the well-known drivers of oncogenesis. Overexpression and constitutively activated kinase signaling results in continuous phosphorylation and activation of signaling pathways well known to contribute to cell growth and proliferation. Constitutive activation of tyrosine kinase signaling is present in numerous types of cancer; overexpression or mutation of at least 30 different tyrosine kinases has been associated with cancer (Blume-Jensen and Hunter, 2001). Tyrosine kinases phosphorylate many glycolytic enzymes as well as components of the pyruvate dehydrogenase complex, promoting the Warburg Effect, increased lactate production and increased tumor growth (Hitosugi et al., 2009, 2011, 2013; Fan et al., 2011, 2014; Shan et al., 2014). In this review, we will summarize the importance of tyrosine phosphorylation of glycolytic enzymes, including phosphoglycerate mutase 1 (PGAM1), lactate dehydrogenase A (LDHA), and pyruvate kinase M2 isoform (PKM2). Tyrosine phosphorylation of PKM2 results in an interesting, paradoxical effect, where phosphorylation decreases PKM2 activity, and this decrease in activity promotes increased glycolytic flux and lactate production in cancer (Hitosugi et al., 2009). In addition to discussing the role of tyrosine kinases in the regulation of the Warburg Effect, we will also summarize the recent studies examining the importance of PKM2 in promoting tumor cell proliferation and tumor growth.

## Tyrosine kinase signaling in the warburg effect

Aberrant tyrosine kinase signaling is a key driver of oncogenesis and tumor growth in numerous different cancers, including both blood cancers and solid tumors. BCR-ABL fusion, TEL-PDGFRβ fusion, FLT3 internal tandem duplication mutation, and JAK2 V617F mutation are all known to contribute to leukemia while FGFR3 mutations are frequently observed in multiple myeloma (Blume-Jensen and Hunter, 2001; Levis and Small, 2003; Renneville et al., 2008). In solid tumors, ErbB2/HER2 overexpression is a well-known driver of breast cancer as well as colon cancer, and EGFR overexpression is commonly observed in lung cancer and head and neck cancers (Blume-Jensen and Hunter, 2001; Baselga, 2006). In addition to the roles of aberrant tyrosine kinase signaling in regulating pathways that promote cell growth and proliferation, tyrosine kinase signaling also influences cellular metabolism (Blume-Jensen and Hunter, 2001; Hitosugi and Chen, 2014). Tyrosine kinase signaling in cancer metabolism functions to enhance the Warburg Effect via increasing glycolysis and lactate production (Hitosugi and Chen, 2014). Glycolytic targets of tyrosine kinase signaling include PGAM1, PKM2, and LDHA where phosphorylation of each of these enzymes promotes increased glycolytic rate and increased tumor cell proliferation (Hitosugi et al., 2009, 2013; Fan et al., 2011).

### Tyrosine phosphorylation of PGAM1

**Figure 1:**
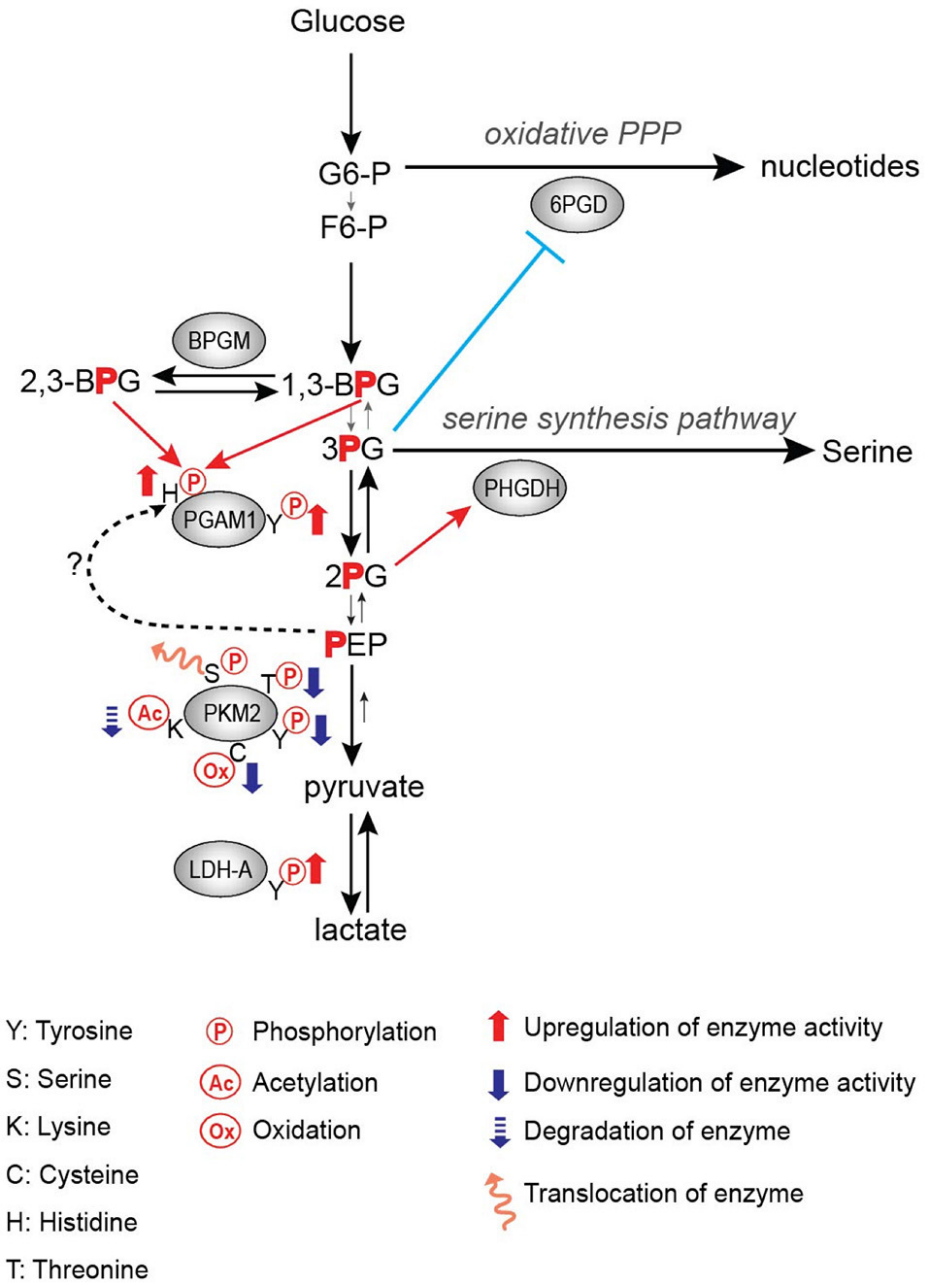
Regulation of glycolytic enzymes by post-translational modifications in the Warburg Effect.

Frequently, tyrosine phosphorylation increases the activity of glycolytic enzymes in cancer. PGAM1 is one such target of tyrosine phosphorylation that displays increased activity upon phosphorylation (Hitosugi et al., 2013). PGAM1 catalyzes the conversion of 3-phosphoglycerate (3-PG) to 2-phosphoglycerate (2-PG) upon binding of the cofactor 2,3-bisphoshoglycerate (2,3-BPG) (Grisolia and Cleland, 1968), and it displays increased expression in hepatocarcinoma and leukemia (Ren et al., 2010; Hitosugi et al., 2012). Consistent with increased expression in promoting tumor growth, phosphorylation of PGAM1 at Y26 by FGFR1 and other tyrosine kinases increases the binding of the cofactor 2,3-BPG to enhance PGAM1 activity and subsequently increasing tumor growth (Figure 1; Hitosugi et al., 2013).

PGAM1 is an important step in the regulation of not only glycolysis, but also branching pathways from glycolysis such as the pentose phosphate pathway (PPP) and the serine biosynthesis pathway. It has been shown that the PGAM1 substrate 3-PG binds to and inhibits 6-phosphogluconate dehydrogenase (6-PGD) in the PPP while the PGAM1 product 2-PG activates 3-phosphoglycerate dehydrogenase (PHGDH) in the serine biosynthesis pathway (Figure 1; Hitosugi et al., 2012). Therefore, PGAM1 inhibition, which increases 3-PG levels and decreases 2-PG levels, decreases PPP and serine biosynthesis fluxes, respectively (Figure 1; Hitosugi et al., 2012). These studies highlight the importance of PGAM1 and its tyrosine phosphorylation in the regulation of glycolysis as well as flux through anabolic biosynthetic pathways to support cancer cell proliferation and tumor growth.

### Tyrosine phosphorylation of LDHA

LDHA catalyzes the conversion of pyruvate to lactate while also regenerating NAD^+^ from NADH. Lactate has numerous proposed roles in promoting tumor growth, including acidifying the tumor microenvironment to promote metastasis and immune invasion, being an energy source for tumor cells, and altering gene expression through its role in regulating transcription factors such as HIF-1 (Chen et al., 2016; Faubert et al., 2017; Brooks, 2018). Thus, the importance of LDHA expression in cancer has been demonstrated in numerous studies, where knockdown or inhibition of LDHA impedes tumor growth (Fantin et al., 2006; Xian et al., 2015; Boudreau et al., 2016). Additionally, LDHA expression is increased in multiple types of cancer, likely driven by c-myc and HIF-1 overexpression (Shim et al., 1997; Kim et al., 2004). Fan et al. showed that tyrosine phosphorylation of LDHA is an additional approach by which oncogenes upregulate LDHA activity to promote tumor growth in non-small cell lung carcinoma (NSCLC) H1299 cell line xenograft model, where phosphorylation of LDHA at Y10 promotes the formation of the highly active tetrameric conformation of LDHA while phosphorylation of Y83 promotes increased binding affinity of LDHA for the cofactor NADH (Figure 1; Fan et al., 2011).

However, contrary to the proposed importance of increased LDHA activity in cancer, LDHA has also been identified as dispensable for tumor growth in lymphoma and brain tumor models (Nilsson et al., 2012; Sundstrom et al., 2015). Additionally, cells can acquire resistance to LDHA inhibition mediated through the AMPK-S6K pathway and an increased ability to utilize oxidative phosphorylation (Boudreau et al., 2016). Thus, the importance of LDHA and tyrosine phosphorylation of LDHA in promoting tumor growth appears to be dependent on the cellular context.

### Tyrosine phosphorylation of PKM2

Activation of glycolytic enzymes via phosphorylation by tyrosine kinases, as observed with PGAM1 and LDHA, logically contributes to increased glycolytic flux and lactate production. However, contrary to the activating effects of tyrosine phosphorylation on PGAM1 and LDHA, phosphorylation of PKM2 results in decreased activity (Hitosugi et al., 2009). PKM2 catalyzes the formation of pyruvate and ATP from phosphoenolpyruvate (PEP) and ADP. Phosphorylation of PKM2 Y105 by tyrosine kinases such as FGFR1, BCR-ABL, and Jak2 inhibits the formation of the highly active tetrameric conformation, thus resulting in decreased PKM2 enzymatic activity (Figure 1; Hitosugi et al., 2009). Inhibition of FGFR1 by the FGFR1 inhibitor TKI258 results in decreased PKM2 Y105 phosphorylation in H1299 cells expressing FGFR1 and in KG-1a cells expressing FOP-FGFR1 fusion (Hitosugi et al., 2009). Paradoxically, this decrease in PKM2 activity promotes increased lactate production and tumor growth (Hitosugi et al., 2009). Thus, PKM2 has continued to be an area of active research to further understand its role in tumorigenesis and cancer cell proliferation.

## PKM2 paradox in the warburg effect

### PKM2 regulation

As one of the irreversible enzymes of glycolysis (K_eq_ approximately 10^4^), pyruvate kinase is thought to be one of the rate limiting steps of glycolysis and thus important in regulating glycolytic activity (Mellati et al., 1992; Christofk et al., 2008b; Nelson et al., 2008). However, whether pyruvate kinase is a rate limiting step in cancer remains under debate (Xie et al., 2016). PKM2 is one of the four pyruvate kinase isoforms. The four pyruvate kinase isoforms are (1) PKL which is primarily expressed in the liver and kidneys, (2) PKR which is exclusively expressed in red blood cells, (3) PKM1 which is highly expressed in differentiated tissues with high energetic demands, and (4) PKM2 which is highly expressed in undifferentiated tissues as well as rapidly proliferating tissues including cancer (Jurica et al., 1998). PKM mRNA is alternatively spliced to produce PKM1 or PKM2, where mutually exclusive selection of exon 9 or 10 results in the expression of PKM1 or PKM2, respectively (David et al., 2010). C-myc drives the expression of polypyrimidine tract binding protein (PTB), heterogeneous nuclear ribonucleoprotein A1 (hnRNPA1) and A2 (hnRNPA2) which function to inhibit the inclusion of exon 9, thus promoting the inclusion of exon 10 and subsequent PKM2 expression (David et al., 2010). SRSF3, a splicing factor that is overexpressed in numerous different cancers, also is capable of binding the PKM transcript and promoting the inclusion of exon 10 to promote PKM2 expression (Wang et al., 2012).

### Low pyruvate kinase activity is important for tumor growth

The relationship between PKM2 expression and activity in cancer has been the focus of numerous studies seeking to elucidate the role of pyruvate kinase activity in regulating tumorigenesis and tumor growth. Because PKM2 has displayed both tumor promoting and tumor suppressive effects, and its activity is in general downregulated in cancer, we have categorized these studies, based on whether decreased pyruvate kinase activity promotes tumor growth or not (Table 1). Eigenbrodt et al. first described the decrease in pyruvate kinase activity with PKM2 expression in transformed cells, noting the paradox between decreased PKM2 activity yet increased glycolysis in the Warburg Effect (Eigenbrodt and Glossmann, 1980) Almost 30 years later, Christofk et al. demonstrated that in H1299 cells, which predominantly express PKM2, stable expression of PKM1 in place of PKM2 resulted in increased pyruvate kinase activity and increased oxidative phosphorylation, yet decreased lactate production and decreased tumor growth (Christofk et al., 2008a). Additional studies also identified PKM2 as a phosphotyrosine binding protein, where binding of phosphotyrosine residues results in decreased pyruvate kinase activity, increased tumor growth, and increased lactate production (Christofk et al., 2008b). These studies show consistent evidence of the PKM2 paradox: decreased pyruvate kinase activity supports increased glycolytic activity and tumor growth.

**Table 1:** Effects of altered PKM2 activity or expression on tumor growth.

**Cancer tissue**	**Oncogenic driver**	**Pyruvate kinase model**	**Tumor growth**
H1299 NCSLC (Christofk et al., 2008a)		mPKM2 as compared to mPKM1 (Low PK activity)	Increased tumor growth
H1299 NCSLC (Hitosugi et al., 2009)	FGFR1	pY105 PKM2 (Low PK activity)	Increased tumor growth
A549 NSCLC (Anastasiou et al., 2011)	ROS	oxC358 PKM2 (Low PK activity)	Increased tumor growth
A549 NSCLC (Yu et al., 2013)	PIM2	pT454 PKM2 (Low PK activity)	Increased cell proliferation
H1299 NSCLC (Lv et al., 2011)		acK305 PKM2 (Degradation)	Increased tumor growth
Breast cancer (Israelsen et al., 2013)	Brca1^fl/fl^ MMTV-Cre Trp53^+/−^	PKM2^Δ/Δ^	Increased tumor growth
Medulloblastoma (Tech et al., 2017)	ND2:SmoA1	PKM2^CKO^	Increased tumor growth
Hepatocellular Carcinoma (Dayton et al., 2016a)		Germline PKM2^−/−^	Increased tumor growth
Leukemia (Wang Y. H. et al., 2014)	BCR-ABL MLL-AF9	PKM2^−/−^	Delayed tumor initiation
Sarcoma (Dayton et al., 2018)	Kras^LSL−G12D/+^;p53^fl/fl^	PKM2^−/−^	Delayed tumor initiation but no effect on tumor growth
Colon cancer (Lau et al., 2017)	APC^CKO^	PKM2^Δ/Δ^	No effect on tumor growth
87-5 SCLC Lu139 SCLC (Morita et al., 2018)		mPKM2	Decreased tumor growth

In addition to the oncogenic drivers that regulate the expression of PKM2, PKM2 activity is regulated via post-translational modifications (Figure 1; Hitosugi et al., 2009; Anastasiou et al., 2011; Lv et al., 2011; Yu et al., 2013; Iansante et al., 2015). Unlike PKM1 which exists in a stable, highly active tetrameric conformation, PKM2 is allosterically regulated by the binding of fructose-1,6-bisphosphate (FBP). The binding of FBP to the low activity dimer confirmation promotes the tetramerization of PKM2, resulting in the formation of the highly active conformation (Jurica et al., 1998). Multiple residues of PKM2 are capable of being post-translationally modified, including tyrosine phosphorylation, serine/threonine phosphorylation, cysteine oxidation, and lysine acetylation (Prakasam et al., 2018). Tyrosine phosphorylation of PKM2 Y105 disrupts FBP binding to inhibit the formation of the highly active tetramer conformation, thus decreasing its enzymatic activity (Hitosugi et al., 2009). This phosphorylation is negatively regulated by protein tyrosine phosphatase 1B (PTP1B), where decreased PTP1B activity results in increased PKM2 Y105 phosphorylation and decreased PKM2 activity (Bettaieb et al., 2013). Threonine phosphorylation of PKM2 T454 by PIM2 functions to inhibit the enzymatic activity of PKM2. Similarly to the observed consequences of Y105 phosphorylation, T454 phosphorylation promotes increased glucose consumption, increased lactate production, and increased cell proliferation (Yu et al., 2013). Cysteine oxidation of C358 upon elevated levels of reactive oxygen species (ROS) also functions to block the formation of the highly active tetramer conformation and inhibit PKM2 activity to promote tumor growth (Anastasiou et al., 2011). Lysine acetylation of PKM2 inhibits PKM2 activity by both decreasing the affinity for the substrate PEP as well as decreasing PKM2 protein stability, which again contributes to tumor growth (Lv et al., 2011). Phosphorylation of PKM2 at T365 by JNK1 results in increased PKM2 activity by increasing the affinity of PKM2 for the substrates PEP and ADP; however, in cancer JNK1 is inactivated by PARP14, thus maintaining the dephosphorylated T365 PKM2 and low activity (Iansante et al., 2015). Again, the expression of PARP14, and subsequent decrease in PKM2 T365 phosphorylation and activity promotes increased glucose consumption and increased lactate production to promote the Warburg Effect (Iansante et al., 2015). Despite the different mechanisms of action in reducing PKM2 activity, each study showed that decreased PKM2 activity via post-translational modification supported the Warburg Effect phenotype and increased tumor proliferation (Table 1; Hitosugi et al., 2009; Anastasiou et al., 2011; Lv et al., 2011; Yu et al., 2013).

Because of the observed importance of decreased PKM2 activity on tumor proliferation, PKM2 activators have been developed as an approach to target cancer. The small molecule PKM2 activators DASA-58 and TEPP-46 were shown to activate PKM2 by promoting the formation of tetrameric PKM2 (Anastasiou et al., 2012). When tested in mouse xenograft models, TEPP-46 treatment resulted in a significant decrease in tumor growth at concentrations that did not cause any major toxicities (Anastasiou et al., 2012). In the clinic, these activators have been tested for the treatment of diseases related to pyruvate kinase deficiency. However, no cancer clinical trials have been completed due to difficulties in selecting the appropriate patient population, as the role of pyruvate kinase activity in cancer is heavily context and tumor dependent.

To further examine the role of PKM2 in promoting cancer cell proliferation, numerous mouse models have been constructed. These models have illustrated an interesting and complicated relationship between PKM2 activity and tumor growth. Some PKM2 deletion models have shown that decreased pyruvate kinase activity increases tumorigenesis. Using a Brca1^fl/fl^ MMTV-Cre Trp53^+/−^ breast cancer model to assess the role of PKM2 in breast cancer tumorigenesis, Israelsen et al. showed that PKM2 specific knockout promoted more rapid breast cancer development (Israelsen et al., 2013). This model allowed for the continued transcription of PKM1 from the PKM gene, and low levels of PKM1 expression were observed in PKM2 ^Δ/Δ^ cells. Despite the low expression of PKM1, the authors concluded that low pyruvate kinase activity was maintained, and this low pyruvate kinase activity is beneficial for tumor growth (Israelsen et al., 2013). This observation is consistent with the published *in vitro* cell line studies (Christofk et al., 2008a; Hitosugi et al., 2009; Anastasiou et al., 2011; Lv et al., 2011). Similar results were observed with a germline PKM2 deletion as well as a medulloblastoma model with PKM2 deletion, where PKM2 ^−/−^ mice displayed increased incidence of hepatocellular carcinoma and increased medulloblastoma tumor growth respectively (Dayton et al., 2016a; Tech et al., 2017). Finally, in a PKM2^fl/fl^ Cre-ER MEF model, the PKM2^Δ/+^ MEFs that gained PKM1 expression displayed slower proliferation than PKM2^*fl*/+^ MEFs (Lunt et al., 2015). These models continue to support the role of decreased PKM2 activity in supporting tumor proliferation (Table 1).

The physiological benefit of this decreased activity continues to be the focus of numerous studies. While it may seem counterintuitive for cancer to display decreased PKM2 activity in the Warburg Effect, it has been proposed that this decrease in PKM2 activity promotes an increase in flux of glycolytic intermediates to biosynthetic pathways including PPP for nucleotide biosynthesis as well as serine biosynthesis pathways (Eigenbrodt and Glossmann, 1980; Anastasiou et al., 2011; Lunt and Vander Heiden, 2011; Lunt et al., 2015). Decreased PKM2 activity has also been proposed to support the increase in an alternative glycolytic pathway. The increase in the levels of the PKM2 substrate PEP caused by decreased PKM2 activity leads to phosphorylation of PGAM1 at histidine 11 (H11), resulting in activation of PGAM1, and thereby further increasing glycolysis and glycolytic intermediates to support macromolecule biosynthesis (Vander Heiden et al., 2010b). In this case, the phosphate group of PEP is transferred to histidine 11 of PGAM1 by an unidentified mechanism (Figure 1; Vander Heiden et al., 2010b). Another recent study has shown that H11 of PGAM1 is non-enzymatically phosphorylated either by 2,3-BPG or 1,3-BPG (Figure 1; Oslund et al., 2017). Since 2,3-BPG levels were increased in PKM2-expressed cells as compared to PKM1-expressed cells (Vander Heiden et al., 2010b), it would be intriguing to examine whether increased H11 phosphorylation by decreased PKM2 activity is a result of a non-enzymatic reaction by increased 2,3-BPG levels or an enzymatic reaction by an unidentified histidine protein kinase (Figure 1). Finally, Cortes-Cros et al. showed that PKM2 knockdown supported an increase in glycolytic biosynthetic intermediates and serine synthesis (Cortes-Cros et al., 2013). They also investigated whether PKM2 regulated glutamine consumption, as glutamine is another major carbon source for anabolic synthesis, and observed that PKM2 knockdown had no effect on glutamine consumption (Cortes-Cros et al., 2013). Thus, decreased PKM2 activity is proposed to support macromolecule biosynthesis through the increased flux of glycolytic intermediates.

### High pyruvate kinase activity is important for tumor growth

There are also models that contradict the importance of decreased PKM2 activity in tumor proliferation. PKM2 inhibition via small molecule inhibitors, such as shikonin, showed that increasing inhibitor concentrations resulted in increased cytotoxicity (Vander Heiden et al., 2010a; Chen et al., 2011; Li et al., 2014). Additionally, curcumin, which has been frequently observed to inhibit cancer cell proliferation, was found to decrease PKM2 expression. This curcumin mediated decrease in PKM2 expression led to decreased glucose consumption, lactate production, and cell proliferation (Siddiqui et al., 2018). Using *in vivo* models, PKM2 deletion in hematopoietic cells delayed the onset of leukemia in BCR-ABL or MLL-AF9 leukemia models (Wang Y. H. et al., 2014). Interestingly, PKM2 inhibition by shikonin, decreased PKM2 expression by curcumin, and PKM2 deletion *in vivo* all resulted in decreased lactate concentrations, which contradict previous models which demonstrated that decreased pyruvate kinase activity increases lactate concentration (Chen et al., 2011; Li et al., 2014; Wang Y. H. et al., 2014; Siddiqui et al., 2018). A recent study by Dayton et al. showed that PKM2 deletion in a Kras^LSL−G12D^/p53 driven sarcoma model results in delayed tumor onset, contradicting the importance of decreased PKM2 activity in tumor initiation (Dayton et al., 2018). However, following tumor initiation, there was no difference in tumor growth between the PKM2^+/+^ and PKM2 ^−/−^ tumors, suggesting PKM2 has no effect on tumor growth (Dayton et al., 2018). Also supporting the notion that PKM2 has no effect on tumor growth, Lau et al. observed no difference in the number of tumors between PKM2^+/+^ and PKM2^Δ/Δ^ mice in an APC^CKO^ colon cancer model (Lau et al., 2017). Finally, Morita et al. observed in small cell lung carcinoma (SCLC) that PKM1 expression, not PKM2, is the isoform responsible for promoting tumor growth (Morita et al., 2018). Thus, these studies contradict the importance of PKM2 in tumor growth (Table 1).

### Non-canonical activities of PKM2

One of the possible explanations to why different cancer types respond differently to changes in pyruvate kinase activity is the non-canonical activity of PKM2. PKM2 has been proposed to be involved in regulating gene transcription through its nuclear and protein kinase activity. PKM2 undergoes nuclear translocation upon interactions with PHD3, JMJD5, and EGFR activation as well as following phosphorylation at PKM2 S37 and S202 by ERK1/2 and Akt respectively (Luo et al., 2011; Yang et al., 2011, 2012b; Wang H. J. et al., 2014; Park et al., 2016). In the nucleus, PKM2 can regulate HIF-1, β-catenin, c-myc, and STAT5 activity, which regulates genes involved in glucose metabolism to promote the Warburg Effect and genes important for supporting increased cell proliferation (Luo et al., 2011; Yang et al., 2011, 2012b; Wang H. J. et al., 2014; Park et al., 2016). While phosphorylation of PKM2 at S37 is important for the translocation of PKM2 into the nucleus from the cytosol, dephosphorylation of PKM2 S37 by Cdc25a in the nucleus is required for the subsequent binding of PKM2 to β-catenin and β-catenin transactivation (Yang et al., 2012b; Liang et al., 2016). Activation of β-catenin promotes increased glucose consumption, increased lactate production, and increased cell proliferation (Liang et al., 2016). Therefore, increased Cdc25a expression and dephosphorylation of PKM2 in the nucleus is important in promoting the Warburg Effect (Liang et al., 2016).

PKM2 is also thought to act as a protein kinase where it uses PEP as a phosphate donor to phosphorylate substrate proteins to regulate gene expression and cell cycle progression (Gao et al., 2012; Yang et al., 2012a; Jiang et al., 2014a,b). However, the protein kinase activity of PKM2 has been debated, and the ability of PKM2 deletion to promote tumor growth further questions the importance of the protein kinase activity of PKM2 in certain cancer models (Israelsen et al., 2013; Hosios et al., 2015). Thus, the roles, or lack thereof, of PKM2 in promoting tumor growth are complex and appear to be heavily context and model dependent.

## Conclusion

Metabolic reprogramming is one of the hallmarks of cancer (Hanahan and Weinberg, 2011). This upregulation of glycolysis and lactate production is a phenotype important for the growth and proliferation of many different types of cancer. Oncogenic tyrosine kinase signaling is one of the key drivers of the Warburg Effect via tyrosine phosphorylation of glycolytic enzymes. Usually, tyrosine phosphorylation results in increased activity of the glycolytic enzyme, consistent with the observed increase in glycolytic flux in the Warburg Effect (Fan et al., 2011; Hitosugi et al., 2013). However, PKM2, the final step in glycolysis, displays decreased enzymatic activity when tyrosine phosphorylated (Hitosugi et al., 2009). This is one example highlighting the PKM2 paradox in cancer: decreased PKM2 activity promotes increased lactate production and tumor growth.

However, not all tumor types or tumor models support the hypothesis that decreased PKM2 activity promotes tumor growth. In a BCR-ABL or MLL-AF9 leukemia model, PKM2 deletion correlates with delayed leukemia onset (Wang Y. H. et al., 2014). In this model, the pyruvate kinase expression level that correlates with the highest lactate concentrations displayed the greatest tumor growth (Wang Y. H. et al., 2014). Thus, despite the opposite effect regarding PKM2 activity, the Warburg Effect holds true: tumor cells display increased glycolysis and increased lactate production.

Questions still remain regarding PKM2 activity, lactate production, and the Warburg Effect. What is not yet clear is how pyruvate kinase activity regulates lactate production. The molecular mechanisms behind the paradox between decreased pyruvate kinase activity and increased lactate production, why the paradox is present in some cancer models and not others, and the role of post-translational modifications of PKM2 via oncogenic tyrosine kinases among others remain to be fully elucidated. The seemingly contradictory effects of PKM2 on cancer cell proliferation and tumor growth also need to be examined further, including whether the effects of PKM2 are dependent on tumor microenvironment, whether PKM2 promotes survival to cellular stress, and whether PKM2 plays a role in promoting metastasis (Dayton et al., 2016b). However, what is becoming increasingly clear is the complexity regarding metabolic regulation in tumor cells, where the regulation of pyruvate kinase activity and isoform expression is important for tumor growth and proliferation.

## Author contributions

All authors listed have made a substantial, direct and intellectual contribution to the work, and approved it for publication.

### Conflict of interest statement

The authors declare that the research was conducted in the absence of any commercial or financial relationships that could be construed as a potential conflict of interest.
